# Supplementary material for: Follow-up focused on psychological intervention initiated after intensive care unit in adult patients and informal caregivers: a systematic review and meta-analysis
Source: PeerJ. 2023 Jun 9;11:e15260. doi: 10.7717/peerj.15260 (PMC10259442; doi:10.7717/peerj.15260)
Supplement: Table S2 [file peerj-11-15260-s002.docx]

**Table S2. Studies without results.**

| Author year | Registry ID Country Observational period | No of patients Intervention/Control | Attrition, % Study status | Type of intervention | Type of intervention against psychological problem | Professionals/sources of intervention | Timing, duration and/or frequency of intervention |
| --- | --- | --- | --- | --- | --- | --- | --- |
| Moulaert et al. 2015 | ISRCTN74835019 Netherlands 12 months after the cardiac arrest | *Caregivers* Total, n=155 No information about sample size in each group  *Patients* 97/88 | *Caregivers* Not stated *Patients* 22.7 | Individualized, semi-structured program for psychological and psychosocial problems | Provision of coping skills | Nurse | From the hospital discharge  Three months, 1-6 times from the first months after discharge |
| Cox et al. 2018 | NCT01983254 The United States of America 12 months after randomization (within two weeks after hospital discharge) | *Caregivers* 86/89 | *Caregivers* 23.3 | Training for psychological problem, combined with Telephone and web | Provision of coping skills | Facilitators other than physician and nurse; Digital media | After hospital discharge  Six telephone sessions for thirty minutes, once per week |
| Haines et al. 2018 | ACTRN12618000615280 Australia 14-17 weeks after hospital discharge | *Patients* Sample size: 60, 1:1 | Recruiting | Participation in group discussion by experience based on co-design | Empathy:  Counselling | Physician; Facilitators other than physician and nurse | From hospital discharge  Two hours per session, total six sessions, once every two weeks, from two-three weeks |
| Khan et al. 2018 | NCT03053245 The United States of America 12 months discharge | *Patients* Sample size: 620, 1:1 | Recruiting | Individualized semi-structured multidisciplinary coordination | Therapy:  Personal care for psychological problem | Nurse; Physician; Facilitators other than physician and nurse | After hospital discharge  Two contacts (within two weeks at hospital discharge), once per two weeks during the first six months, and once a month during the last six months |
| Malik et al. 2018 | NCT03431493 The United States of America 12 weeks after hospital discharge | *Patients* Sample size: 54, 1:1 | Recruiting | Rehabilitation for physical and psychological problem | Therapy:  Cognitive behavioral therapy | Facilitators other than physician and nurse | After hospital discharge  Within one week |
| Rohr et al. 2021 | NCT04186468 Germany Six months after ICU discharge | *Patients* Sample size: 100, 1:1 | Recruiting | Establishment of a network of health care professionals and consultation with experts for physical, mental, cognitive and social functional problem | Psychological intervention required after monitoring | Physician; Facilitators other than physician and nurse | After ICU discharge  Consultation at 1-3 times from about 2 months |
| Ewens et al. 2019 | PRR1-10.2196/10935 Australia 12 months after hospital discharge | *Patients* Sample size: 162, 1:1 | Not detail | Self-directed multidisciplinary monitoring and advice | Psychological intervention required after monitoring | Digital media | Desired frequency of visit with website |
| Boehm et al. 2019 | NCT03926533 The United States of America 6 months after hospital discharge | *Patients* Sample size: 120, 1:1 | Recruiting | Telephone-based multidisciplinary case management for PICS | Psychological intervention required after monitoring | Not detail | After hospital discharge  Ninety minutes per time, two times at three weeks and three months |
| Ojeda et al. 2021 | NCT04394169 Spain 6 months after hospital discharge | *Patients* Sample size: 102, 1:1 | Recruiting | Individualized, semi-structured program for PICS | Therapy:  Cognitive behavioral therapy | Not detail | After hospital discharge  Three times visits at Four-six, eight, and 18 weeks Psychological intervention was 7 weekly sessions for 90 minutes per one session |
| Cox et al. 2020b | NCT04329702 The United States of America 3 months after discharge | *Patients* Sample size: 45, 1:1 | Recruiting | (1) Self-directed training with call from therapist for psychological problem | (1) Provision of coping skills | (1) Facilitators other than physician and nurse; Digital media | After hospital discharge  Four weeks |
|  |  |  |  | (2) Self-directed training for psychological problem | (2) Provision of coping skills | (2) Digital media |  |
| Gawlytta et al. 2020 | DRKS00010676 Germany 1 month after ICU discharge | *Patients and caregivers* 12/13 (Units) | Not detail | Web-based therapy for psychological problem | Therapy:  Cognitive behavioral therapy | Digital media | From ICU discharge  50 minutes per week, four weeks from at least one month |
| Chen et al. 2022 | DOI 10.17605/ OSF.IO/PD7SU China 2 weeks after ICU discharge | *Patients* Sample size: 110, 1:1 | Not detail | Mindfulness-based training to reduce stress | Provision of coping skills | Nurse; Physician; Facilitators other than physician and nurse | After ICU discharge  Two weeks |
| Friedman et al. 2022 | NCT01796509 French 12 months after ICU discharge | *Patients* Sample size: 546, 1:1 | Not detail | Multidisciplinary consultation | Empathy:  Counselling | Physician; Psychologist; Social worker | After ICU discharge  First week, 3 months, 6 months |
