# Supplementary material for: Follow-up focused on psychological intervention initiated after intensive care unit in adult patients and informal caregivers: a systematic review and meta-analysis
Source: PeerJ. 2023 Jun 9;11:e15260. doi: 10.7717/peerj.15260 (PMC10259442; doi:10.7717/peerj.15260)
Supplement: Table S3 [file peerj-11-15260-s003.docx]

**Table S3. Excluded studies list.**

| First investigator | Title | Registory ID | Main reason for exclusion | Publication |
| --- | --- | --- | --- | --- |
| Gensichen et al. | Effect of a combined brief narrative exposure therapy with case management versus treatment as usual in primary care for patients with traumatic stress sequelae following intensive care medicine: study protocol for a multicenter randomized controlled trial (PICTURE) | NCT03315390 | Intervention not initiated within one month after hospital discharge | N |
| Faux et al. | Does very early therapy reduce the time spent hospital and improve the recovery of people with seriousillness? | ACTRN12618000539235 | Just enhanced physical rehabilitation compared with usual care | N |
| Bannon et al. | Recovering together: building resiliency in dyads of stroke patients and their caregivers at risk for chronic emotional distress; a feasibility study | NCT02797509 | Initiation of intervention before ICU discharge | N |
| Bates et al. | CovEMERALD: Assessing the feasibility and preliminary effectiveness of remotely delivered Eye Movement Desensitisation and Reprocessing following Covid-19 related critical illness: A structured summary of a study protocol for a andomized controlled trial | NCT04455360 | Unclear initiation of intervention | N |
| Elliott et al. | Health-related quality of life and physical recovery after a critical illness: a multi-centre andomized controlled trial of a home-based physical rehabilitation program | ACTRN12605000166673 | Just enhanced physical rehabilitation compared with usual care | Y |
| Batterham et al. | Effect of supervised aerobic exercise rehabilitation on physical fitness and quality-of-life in survivors of critical illness: an exploratory minimized controlled trial (PIX study) | ISRCTN65176374 | Just enhanced physical rehabilitation compared with usual care | Y |
| Jones et al. | Improving rehabilitation after critical illness through outpatient physiotherapy classes and essential amino acid supplement: A randomized controlled trial | NCT01063738 | Provision of coping skill for mental problems in control group | Y |
| Kalra et al. | Training carers of stroke patients: andomized controlled trial | Not stated | Population without need of intensive care | Y |
| Hickman et al. | Self-Management Behaviors of Caregivers of the Chronically Critically Ill (ASSIST) | NCT03065829 | Unclear detail of intervention | N |
| Mudry et al. | A Recovery-Oriented Counselling Group | NCT03925181 | Unclear initiation of intervention | N |
| Bakhru et al. | Wake Forest Post-ICU Telehealth (WFIT) Program (WFIT) | NCT04576065 | Unclear initiation of intervention | N |
| Cox et al. | Lift Mobile Mindfulness for COVID-19 Distress Symptoms (LIFTCOVID) | NCT04581200 | Inclusion of hospitalized patients | N |
| Petrinec et al. | Smartphone Delivery of Cognitive Behavioral Therapy for Postintensive Care Syndrome-Family: Protocol for a Pilot Study | NCT04316767 | Initiation of intervention before ICU discharge | N |
| Børøsund et al. | The Caregiver Pathway – An Intervention to Support Caregivers of Critically Ill Patients | NCT04839406 | Initiation of intervention before ICU discharge | N |
| Connor et al. | Expect the unexpected: clinical trials are key to understanding post-intensive care syndrome | Not stated | Secondary analysis of RCT | Y |
| McDowell et al. | Effectiveness of an exercise programme on physical function in patients discharged from hospital following critical illness: a andomized controlled trial (the REVIVE trial) | NCT01463579 | Just enhanced physical rehabilitation compared with usual care | Y |
| Orwelius et al. | Evaluate efficacy of tele‑yoga on multiple outcomes after a period of critical illness | Not stated | Yoga is not covered in this SR. | Congress report |
| Sluisveld et al. | A strategy to enhance the safety and efficiency ofhandovers of ICU patients: study protocol of thepICUp study | Not stated | A descriptive, explorative study using a mixed method design | N |
| Vitacca et al. | Does 6-Month Home Caregiver-Supervised Physiotherapy Improve Post-Critical Care Outcomes?: A Randomized Controlled Trial | NCT01577927 | Just enhanced physical rehabilitation compared with usual care | Y |
| Vlake et al. | Effect of intensive care unit-specific virtualreality (ICU-VR) to improve psychologicalwell-being and quality of life in COVID-19ICU survivors: a study protocol for amulticentre, randomized controlled trial | NL8835 | Initiation of intervention from either 3 or 6 months after hospital discharge | N |
| Smith et al. | A randomized trial to evaluate an education programme for patients and carers after stroke | Not stated | Population without need of intensive care | Y |
| Eames et al. | Randomised controlled trial of aneducation and support package forstroke patients and their carers | ACTRN12608000469314 | Population without need of intensive care | Y |
| Fernandes et al. | Brief psychological intervention in phase I of cardiac rehabilitation after acute coronary syndrome | Not stated | Population without need of intensive care | Y |
| Khandelwal et al. | Prevalence, Risk Factors, and Outcomes of Financial Stress in Survivors of Critical Illness | Not stated | Secondary analysis of RCT | Y |
| Känel et al. | Early Psychological Counseling for the Prevention of Posttraumatic Stress Induced by Acute Coronary Syndrome: The MI-SPRINT Randomized Controlled Trial | NCT0178124 | Population without need of intensive care | Y |
| Carvalho et al. | Therapeutic respiratory and functional rehabilitation protocol for intensive care unit patients affected by COVID-19: a structured summary of a study protocol for a andomized controlled trial | RBR-7rvhpq9 | Just enhanced physical rehabilitation compared with usual care | N |
| Connolly et al. | Exercise-based rehabilitation after hospital discharge for survivors ofcritical illness with intensive care unit–acquired weakness: A pilotfeasibility trial | NCT00976807 | Just enhanced physical rehabilitation compared with usual care | Y |
| Cox et al. | Optimizing a self-directed mobile mindfulness intervention for improving cardiorespiratory failure survivors’ psychological distress (LIFT2): Design and rationale of a randomized factorial experimental clinical trial | NCT04038567 | Self-directed training for psychological problem in control group | N |
| Castillo et al. | Well-being and medical recovery in the Critical Care Unit: The role of the nurse-patient interaction | Not stated | Initiation of intervention at andomize room | Y |
| Hamilton et al. | Determinants of depressive symptoms at one year after intensive care unit (ICU) discharge in survivors of ≥ seven days of mechanical ventilation: results from the recover program | Not stated | A secondary analysis of a prospective multicenter cohort study | Y |
| Hoffmann et al. | Anxiety, depression and stress in relatives of intensive care unit patients-baseline data of a randomized controlled trial | Not stated | A secondary analysis of a RCT for andom support in the ICU (NCT02931851) | Congress report |
| Arab et al. | Assessing the impact of using a self help rehabilitation program on post ICU patient’shealth status in university hospitals of Kerman University of Medical Sciences. | IRCT201110197844N1 | Unclear detail of intervention | N |
| Alboativi et al. | Investigating the Impact of Nursing Nursing Transmission from ICUs to General Sectorson Anxiety and Satisfaction of Patients and Their Families | IRCT20180210038685N1 | Unclear detail of intervention | N |
| Preller et al. | Cognitive behavioural therapy (CBT) for the treatment of post-traumatic stress disorder(PTSD) in intensive care unit (ICU) survivors | ISRCTN97280643 | A single-centre observational treatment case-control study | N |
| Jensen et al. | Towards a new orientation: a qualitative longitudinal study of anintensive care recovery programme | Not stated | A secondary analysis of a included RCT (NCT01721239) | Y |
| Jensen et al. | Intervention fidelity in postintensive care follow-up consultations at ten sites in the RAPIT-trial: A mixed-methods evaluation | Not stated | A secondary analysis of a included RCT (NCT01721239) | Y |
| McGregor et al. | Rehabilitation exercise and psychological support after covid-19 infection (REGAIN): a structured summary of a study protocol for a andomized controlled trial | ISRCTN11466448 | Initiation of intervention from more than three months after discharge | N |
| Unknown, Haukeland University Hospital | Rehabilitation After Intensive Care (REHAB) | NCT01770821 | Unclear detail of intervention | N |
| Jutte et al. | Critical Care Anxiety and Long-Term Outcomes Management (CALM) | NCT02421861 | Unclear detail of intervention | N |
| Walsh et al. | Increased Hospital-Based Physical Rehabilitationand Information Provision After Intensive Care Unit DischargeThe RECOVER Randomized Clinical Trial | ISRCTN09412438 | Provision of coping skill for mental problems in control group | Y |
| Rodgers et al. | Randomized Controlled Trial of a Comprehensive StrokeEducation Program for Patients and Caregivers | Not stated | Population without need of intensive care | Y |
| Walker et al. | Biopsychosocial intervention for stroke carers (BISC): results of a feasibility andomized controlled trial and nested qualitative interview study | ISRCTN15643456 | Population without need of intensive care | Y |
| Wang et al. | Improving Recovery and Outcomes Every Day after the ICU (IMPROVE): study protocol for a randomized controlled trial | NCT03095417 | Intevention forcusd on decline of cognitive function | N |
| Wendlandt et al. | Informing Decisions in Chronic Critical Illness: A Randomized Control Trial (RCT) | NCT01230099 | Familiy support in the ICU | Y |
| Rego et al. | The effect of physical therapy treatment on the quality of life and physical function of adults hospitalized in the Intensive Care Unit | RBR-9wghvc | Initiation of intervention before ICU discharge | N |
| Wu et al. | Can in-reach multidisciplinary rehabilitation in the acute ward improve outcomes for critical care survivors? A pilot randomized controlled trial | ACTRN12618000539235 | Initiation of intervention before ICU discharge | Y |
| Longueville at el. | Evaluation of Psychological Impact of Group Therapy for Patients Who Have Been Hospitalized in Intensive Care During COVID-19 Pandemic: Exploratory Study (GPR COVID) | NCT04747405 | Initiation of intervention before ICU discharge | N |
| Gilmartin et al. | Intensive care discharge facilitation using the Rehabilitation after Critical illness Assisted discharge Pack (RECAP) model: A pilot randomized controlled trial | NCT02415634 | Just enhanced physical rehabilitation compared with usual care | Y |
| Hellman et al. | Web-based Follow-up to Former ICU Patients (WIVA) | NCT04317144 | Unclear initiation of intervention | N |
| Hickman et al. | Self-Management Behaviors of Caregivers of the Chronically Critically Ill (ASSIST) | NCT03065829 | Intervention of meditation and sleep assist | N |
| Bakhru et al. | Wake Forest Post-ICU Telehealth (WFIT) Program | NCT04576065 | Unclear initiation of intervention | N |
| Sayde et al. | Implementing an intensive care unit (ICU) diary program at a large academic medical center: Results from a randomized control trial evaluating psychological morbidity associated with critical illness | NCT04305353 | Initiation of intervention before ICU discharge, and education for a psychological problem in the control group | Y |
| Karnatovskaia et al. | Positive Suggestions Via MP3 Messages | NCT04437095 | Unclear initiation of intervention | N |
| Mayer et al. | Redefining Survivorship: POWER to Maximize Physical Function for Survivors of Critical Illness (POWER) | NCT04058977 | Just enhanced physical rehabilitation compared with usual care | N |
| Flaatten et al. | Impact on Family or Care-givers of Very Old ICU-survivors, Trajectories and 6 Months’ Outcome in the Very Old. (VIP2) | NCT03138278 | Cluster-randomisation | N |
| Munro et al. | Sensation Awareness Focused Training for Spouses (SAF-T) | NCT03129204 | Initiation of intervention before ICU discharge | N |
| Bérubé et al. | A Hybrid Web-Based and In-Person Self-Management Intervention Aimed at Preventing Acute to Chronic Pain Transition After Major Lower Extremity Trauma: Feasibility and Acceptability of iPACT-E-Trauma | ISRCTN91987302 | Unclear population without need of intensive care | Y |
| Danesh et al. | Peer Support for Post Intensive Care Syndrome Self-Management (PS-PICS) | NCT03788096 | Unclear initiation of intervention | N |
| Skogstad et al. | Nurse-Led Psychological Intervention After Physical Traumas: A Randomized Controlled Trial | Not stated | Patients admitted in a trauma referral center | Y |
| Stayt et al. | Making sense of it: intensive care patients’ phenomenological accounts of story construction | Not stated | A descriptive study of social, psychological, and historical experiences by individuals | Y |
| Bryant et al. | A Randomized Controlled Trial of Exposure Therapy and Cognitive Restructuring for Posttraumatic Stress Disorder | Not stated | Population without need of intensive care | Y |
| Griffith et al. | Determinants of Health-Related Quality of Life After ICU: Importance of Patient Demographics, Previous Comorbidity, and Severity of Illness | Not stated | A secondary analysis of a included RCT (ISRCTN09412438) | Y |
| Kelly et al. | Patients’ recovery after critical illness at early follow-up | Not stated | A descriptive study about patients after intensive care | Y |
| Fulcher et al. | Randomised controlled trial of graded exercise in patients with the chronic fatigue syndrome | Not stated | Population without need of intensive care | Y |
| Jones et al. | Intensive care diaries reduce new onset post traumatic stress disorder following critical illness: a andomized, controlled trial | NCT00912613 | No arm of intervention other than ICU diary | Y |
| Knowles et al. | Evaluation of the effect of prospective patient diaries on emotional well-being in intensive care unit survivors: A randomized controlled trial | Not stated | No arm of intervention other than ICU diary | Y |
| Garrouste-Orgeas et al. | Impact of an intensive care unit diary on psychological distress in patients and relatives | Not stated | No arm of intervention other than ICU diary | Y |
| Egerod et al. | Intensive care patient diaries in Scandinavia: a comparative study of emergence and evolution | Not stated | A andomizedve study combined some datasets | Y |
| Denehy et al. | Exercise rehabilitation for patients with critical illness: a randomized controlled trial with 12 months of follow-up | ACTRN12605000776606 | Just enhanced physical rehabilitation compared with usual care | Y |
| McWilliams et al. | Feasibility and impact of a structured, exercise-based rehabilitation programme for intensive care survivors | Not stated | A case series study | Y |
| Schweickert et al. | Early physical and occupational therapy in mechanically ventilated, critically ill patients: a andomized controlled trial | NCT00322010 | Initiation of just enhanced physical rehabilitation before ICU discharge | Y |
| Jackson et al. | Cognitive and physical rehabilitation of intensive care unit survivors: Results of the RETURN randomized controlled pilot investigation | NCT00715494 | Intevention focused on decline of cognitive function | Y |
| Wade et al. | Effect of a Nurse-Led Preventive Psychological Intervention on Symptoms of Posttraumatic Stress Disorder Among Critically Ill Patients A Randomized Clinical Trial | ISRCTN53448131 | Initiation of intervention before ICU discharge | Y |
| White et al. | A Randomized Trial of a Family-Support Intervention in Intensive Care UnitsA Randomized Trial of a Family-Support Intervention in Intensive Care Units | NCT01844492 | Initiation of intervention before ICU discharge | Y |
| Czerwonka et al. | Changing support needs of survivors of complex critical illness and their family caregivers across the care continuum: A qualitative pilot study of Towards RECOVER | NCT00896220 | A prospective cohort study | Y |
| Hickman et al. | Impact of Chronic Critical Illness on the Psychological Outcomes of Family Members | Not stated | A narrative review of the psychological outcomes of informal caregivers | Y |
| Brummel et al. | A combined early cognitive and physical rehabilitation program for people who are critically ill: the activity and cognitive therapy in the intensive care unit (ACT-ICU) trial | NCT01270269 | Initiation focused on physical and cognitive function before ICU discharge | Y |
| Battle et al. | Supervised exercise rehabilitation in survivors of critical illness: A andomized controlled trial | ISRCTN11853373 | Just enhanced physical rehabilitation compared with usual care | Y |
| Lester et al. | Can a Dyadic Resiliency Program Improve Quality of Life in Cognitively Intact Dyads of Neuro-ICU Survivors and Informal Caregivers? Results from a Pilot RCT | NCT03694678 | Initiation of intervention before ICU discharge | Y |
| Chiang et al. | A Brief Cognitive-Behavioral Psycho-Education (B-CBE) Program for Managing Stress and Anxiety of Main Family Caregivers of Patients in the Intensive Care Unit | Not stated | Initiation of intervention before ICU discharge | Y |
| Stiekema et al. | Case management after acquired brain injury compared to care as usual: study protocol for a two- year pragmatic randomized controlled superiority trial with two parallel groups | NL8104 | Unknown initiation of intervention | N |
| Chiang et al. | Effects of physical training on functional status in patients with prolonged mechanical ventilation | Not stated | Intervention combined physical rehabilitation and diaphragmatic breathing exercise | Y |
| McKinney et al. | Leaving the intensive care unit: a phenomenological study of the patients’ experience | Not stated | A narrative review of the psychological study for patients after intensive care | Y |
| Paul et al. | Meeting patient and relatives’ information needs upon transfer from an intensive care unit: the development and evaluation of an information booklet | Not stated | A descriptive study about interview about an booklet for patients and caregivers from ICU | Y |
| Burtin et al. | Early exercise in critically ill patients enhances short-term functional recovery | Not stated | Intervention of bedside cycle ergometer conducted in ICU | Y |
| Wright et al. | Intensive versus standard physical rehabilitation therapy in the critically ill (EPICC): a multicentre, parallel-group, andomized controlled trial | ISRCTN20436833 | Initiation of intervention before ICU discharge | Y |
| Gruther et al. | Can Early Rehabilitation on the General Ward After an Intensive Care Unit Stay Reduce Hospital Length of Stay in Survivors of Critical Illness? A Randomized Controlled Trial | NCT02754505 | Just enhanced physical rehabilitation compared with usual care | Y |
| McWilliams et al. | Earlier and enhanced rehabilitation of mechanically ventilated patients in critical care: A feasibility andomized controlled trial. | ISRCTN90103222 | Initiation of intervention before ICU discharge | Y |
| Strömberg et al. | Tele-Yoga in Long Term Illness-Protocol for a Randomised Controlled Trial Including a Process Evaluation and Results from a Pilot Study | NCT03703609 | Yoga intervention using telephone | N |
| Bryant et al. | Treating Acute Stress Disorder: An Evaluation of Cognitive Behavior Therapy and Supportive Counseling Techniques | Not stated | Intervention for PTSD in all arms | Y |
| Haines et al. | Feasibility of Virtual Peer Support for ICU Survivors: icuRESOLVE-D (Intensive Care Unit REcovery Solutions cO-Led through surVivor Engagement Digital) Study | ACTRN12621000737831 | Initiation of intervention before ICU discharge | N |
| Dekeyser et al. | Combined psychologist-physician post-death meeting as part of an integrated bereavement program for families | Not stated | A non-randomized study | Y |
