# Supplementary figures and images for: Follow-up focused on psychological intervention initiated after intensive care unit in adult patients and informal caregivers: a systematic review and meta-analysis
Source: PeerJ. 2023 Jun 9;11:e15260. doi: 10.7717/peerj.15260 (PMC10259442; doi:10.7717/peerj.15260)

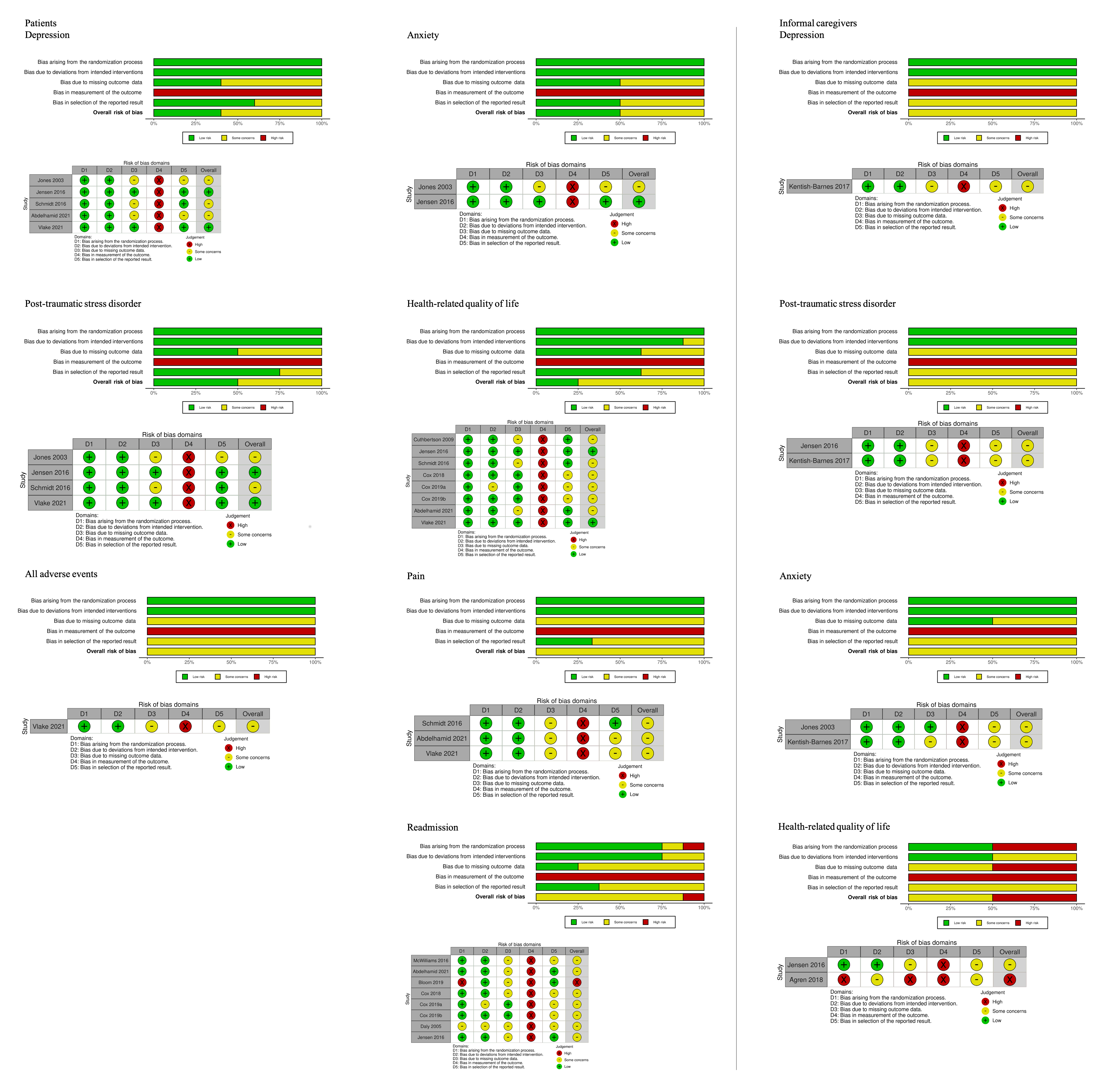

Supplement: Figure S1 [file peerj-11-15260-s004.png]

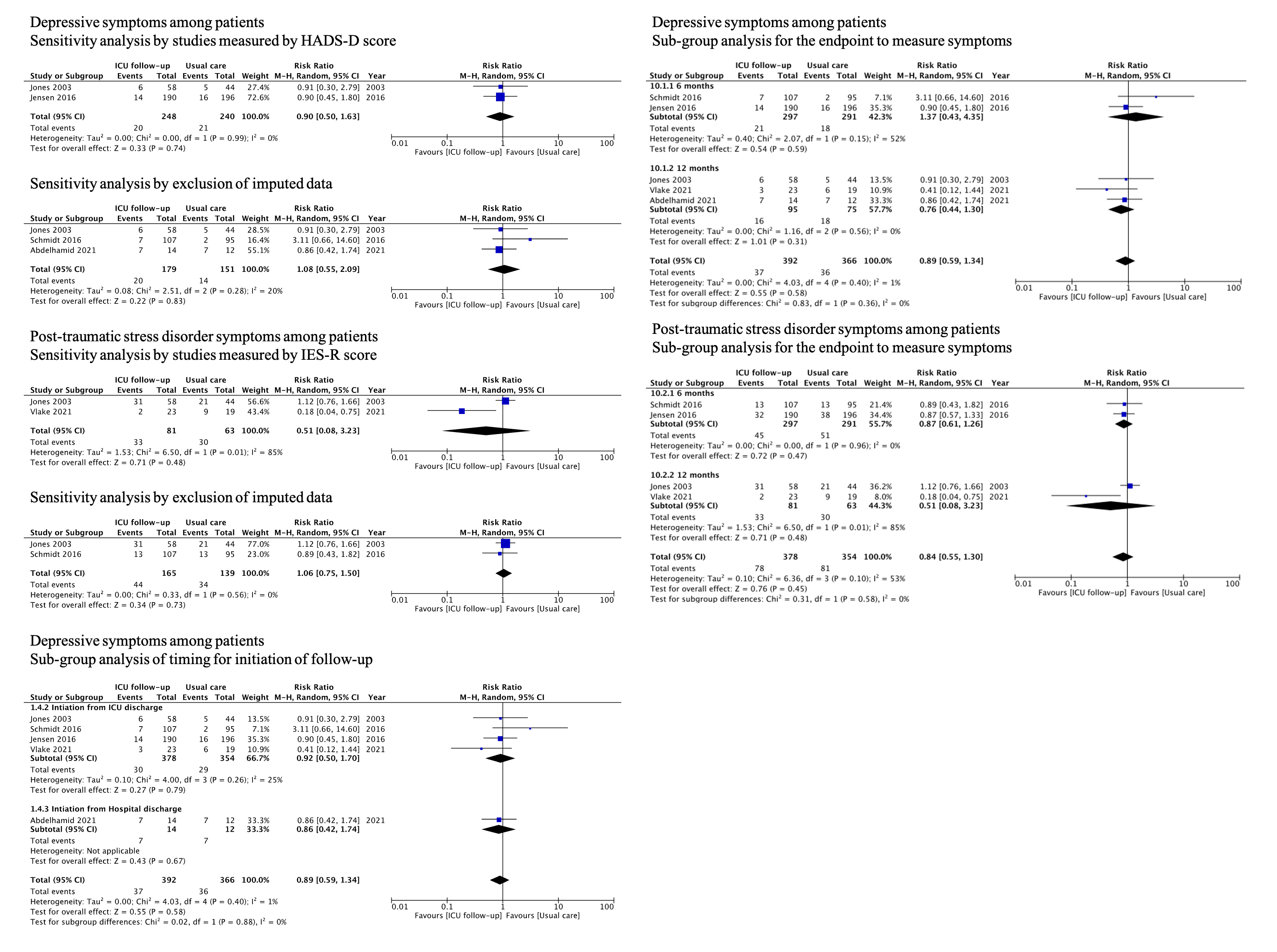

Supplement: Figure S2 [file peerj-11-15260-s005.png]

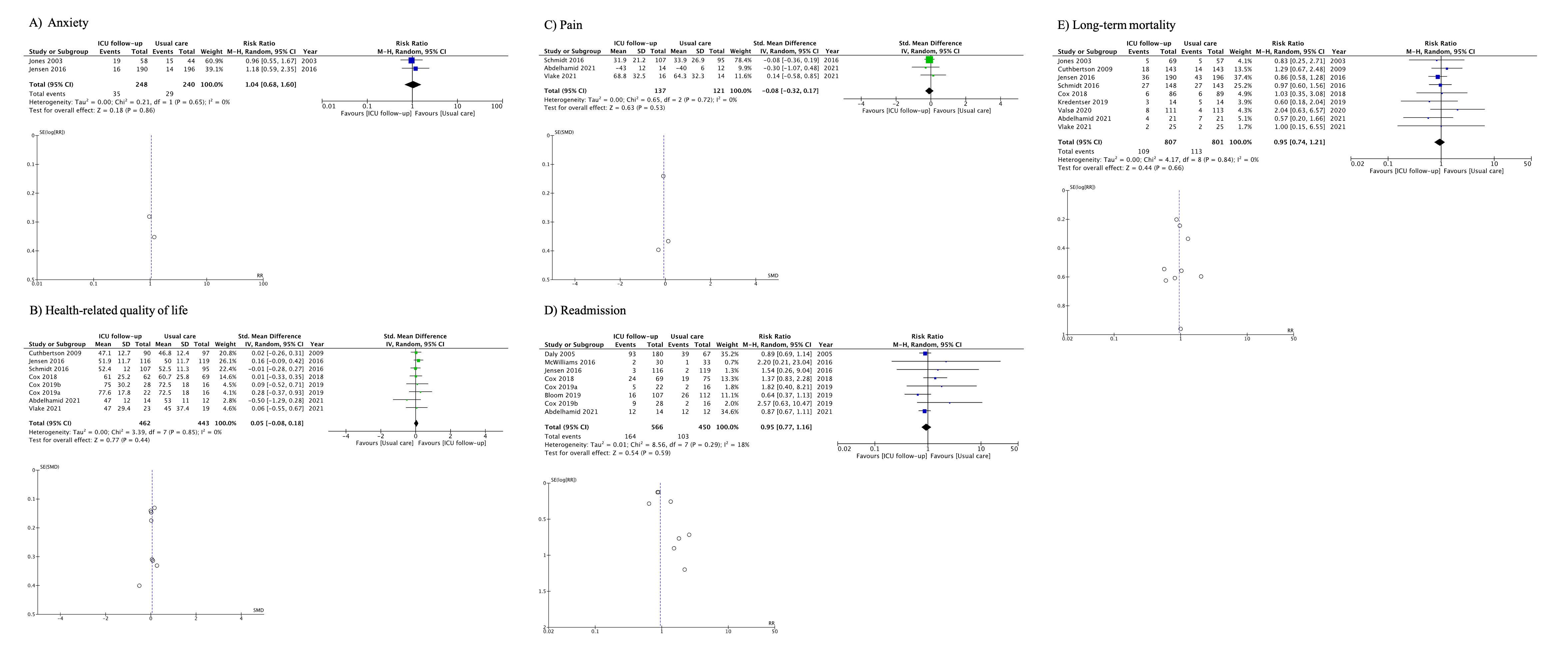

Supplement: Figure S3 [file peerj-11-15260-s006.png]

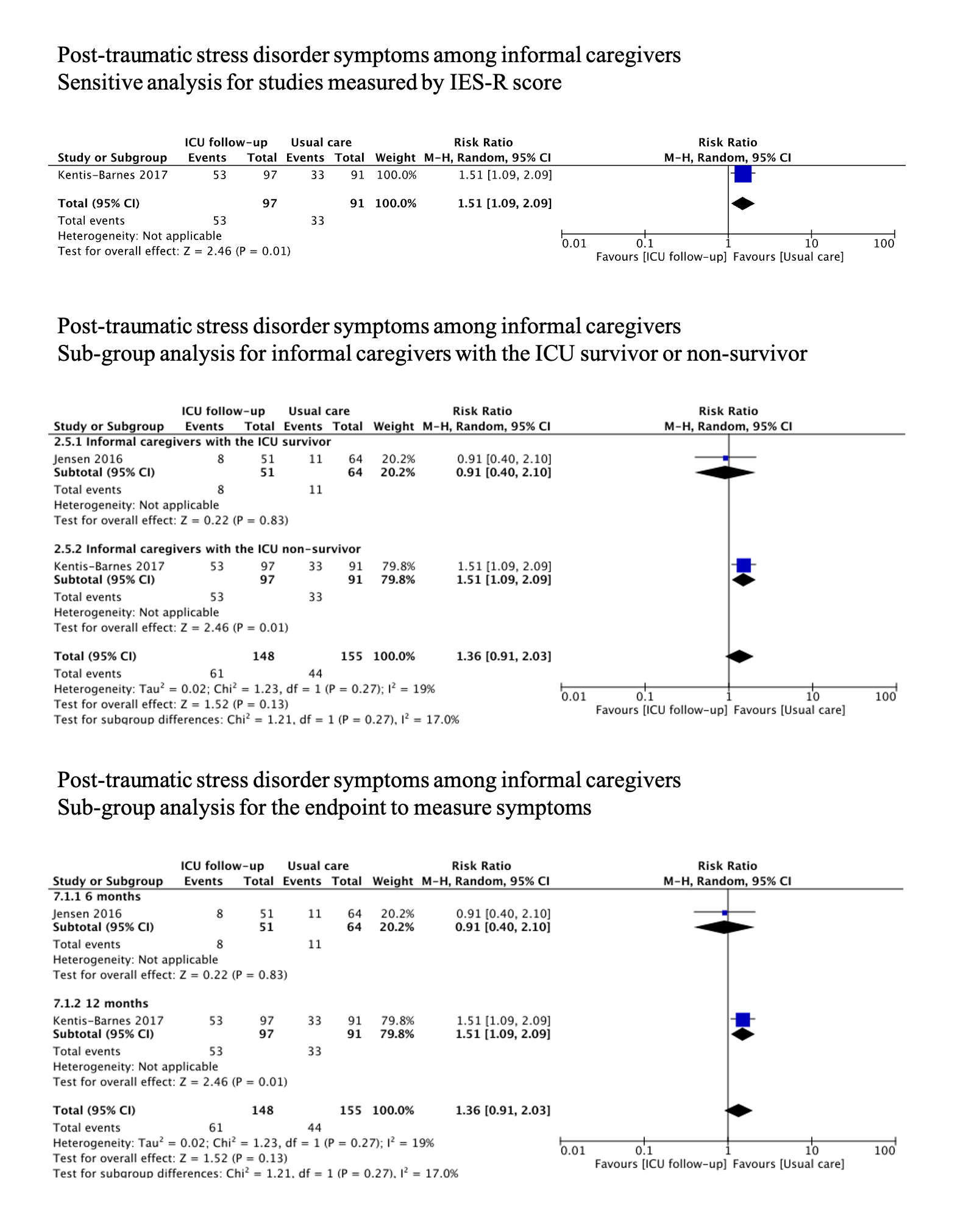

Supplement: Figure S4 [file peerj-11-15260-s007.png]

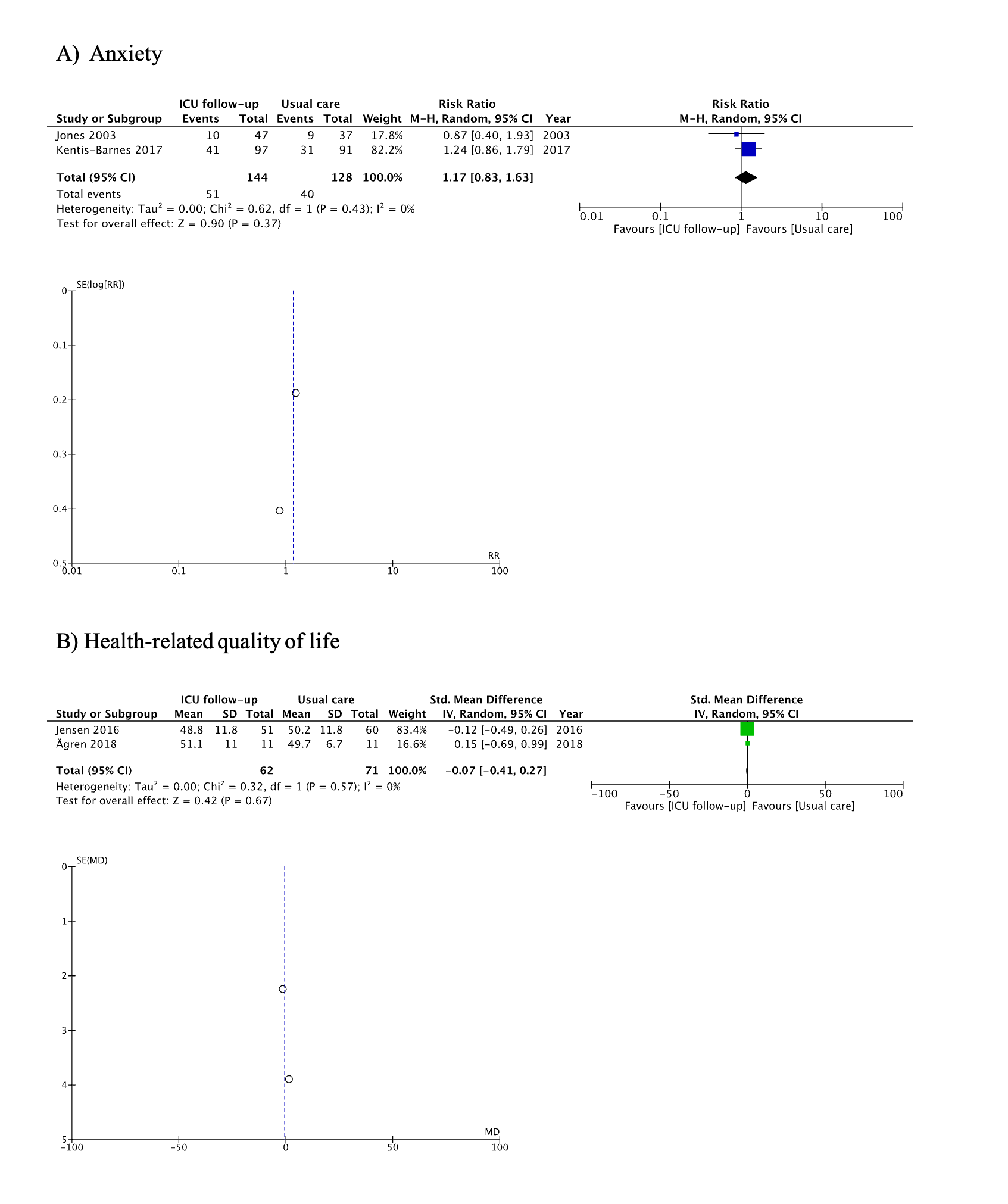

Supplement: Figure S5 [file peerj-11-15260-s008.png]
