# Supplementary material for: Follow-up focused on psychological intervention initiated after intensive care unit in adult patients and informal caregivers: a systematic review and meta-analysis
Source: PeerJ. 2023 Jun 9;11:e15260. doi: 10.7717/peerj.15260 (PMC10259442; doi:10.7717/peerj.15260)
Supplement: Supplemental Information 10 [file peerj-11-15260-s010.docx]

**Follow-up focused on psychological intervention initiated after intensive care unit in adult patients and informal caregivers: A systematic review and meta-analysis**

**1. Rationale for conducting the systematic review**

Adult patients who are admitted to intensive care units (ICU) and their informal caregivers may experience psychological dysfunction, which could persist after discharge. The psychological dysfunction of patients and their informal caregivers was called post-intensive care syndrome (PICS), composed of psychological dysfunction, cognitive impairment, and muscle weakness. According to the current guidelines and a systematic review (SR), follow-up with patients who had been admitted to the ICU comprises a diversity of contents, targets, and timings of initiation. The guideline for follow-ups recommended providing enhanced or individualized physical intervention from early mobilization to home rehabilitation. A recent SR studied psychological intervention for patients’ informal caregivers; however, the SR did not separately investigate adult patients and pediatric patients. In the pediatric randomized controlled trials, interventions were specific for children such as skin-to-skin contact, kangaroo care, or guidance for baby care. There was clinical heterogeneity between included studies in the previous SR. Hence, the effects of follow-ups for adult patients and informal caregivers following ICU discharge and focused on psychological interventions are unknown.

**2. The contribution that the systematic review makes to knowledge in light of previously published related reports.**

The follow-up initiated after ICU discharge and focused on psychological interventions did not reduce psychological dysfunction among critically-ill patients and informal caregivers. Though previous SR suggested favorite outcomes of critically-ill patients and informal caregivers, ICU follow-ups focused on psychological interventions may have the opposite result. and the certainty-of-evidence was low at best. The prevalence of psychological dysfunction among patients in the usual care group in our SR/MA was lower than that among patients in previous reviews. Though studies for patients with a high risk of PICS should be required, such participants might not be able to understand the education to find and avoid psychological intervention by themselves, since symptoms of PICS overlapped. Thus, routine initiation for these follow-ups might be avoided in clinical situations, and studies for follow-ups focusing on other psychological interventions initiated after ICU discharge need to be conducted.
